# Calendar

**DOI:** 10.1155/S146392468100031X

**Published:** 1981


### Calendar

**Editor's Note:**
*Organisers of conferences, seminars etc. should send details for inclusion in this calendar as soon as the relevant information is available and not later than three months before the event.*

**SEMLAB '81**
June 2-5, London
*Industrial Trade Fairs Ltd, Radcliffe House, Blenheim Court, Solihull, West Midlands B91 2BG*

**Precision measurement and fundamental constants, 2nd international conference**
June 8-12, Gaithersburgh, Maryland, USA
*NBS, Bldg 220, Room B258, Washington DC, 20234, USA*

**Chromatography in biochemistry, medicine, and environmental research, 1st international symposium**
June 16-17, Venice
*Alberto Frigerio, Italian Group for Mass Spectrometry in Biochemistry and Medicine, c/o Instituto di Richerche Farmacologiche (Mario Negri), Via Eritrea 62-20157 Milan, Italy*

**Mass spectrometry in biochemistry, medicine, and environmental research, 8th international symposium**
June 18-19, Venice
*Dr Alberto Frigerio, Italian Group for Mass Spectrometry in Biochemistry and Medicine, c/o Instituto di Richerche Farmacologiche (Mario Negri), Via Entrea 62-20157 Milan, Italy*

**Affinity chromatography and related techniques**
June 22-26, Nijmegan, The Netherlands
*T.C.J. Gribnau, Organon Scientific Development Group, PO Box 20, 5340BH, Oss, The Netherlands*

**Clinical Chemistry, XVIII Nordic Congress**
June 23-26, Reykjavik
*XVIII Nordic Congress, Rannsoknadeild, Borgarspitalinn, 108 Reykjavik, Iceland*

**Analytical mass spectrometry**
June 30 - July 2, Pittsburgh
*D.M. Hercules, Dept. of Chemistry, University of Pittsburgh, PA 15260, USA*

**Computers in automation and laboratory management; automatic chemical analysis summer school**
July 5-10, Swansea
*Professor D. Betteridge, Chemistry Dept, University College of Swansea, Singleton Park, Swansea*

**Safety of electrical instrumentation in potentially explosive atmospheres, residential course**
July 7-9, Chislehurst
*Conference and courses Unit, Sira Institute Ltd, South Hill, Chislehurst, Kent*

**5th International NMR Meeting**
July 12-16, Exeter
*D. Shaw, Oxford Research Systems, Ferry Hinksey Road, Oxford OX2 ODF*

**Automatica '81**
August 18-22, Oslo
*Information Officer, Norges Varemesse, Drammensvein 154, PO Box 130, Skoyen, Oslo 2, Norway*

**Euroanalysis IV**
August 23-28, Helsinki, Finland
*Euroanalysis IV, Association of Finnish Chemical Societies, Mr. Veikko Velamo, Executive Director, Pohj Hesperiankatu 3B 10, SF-00260 Helsinki 26, Finland*

**Analytical chemistry, 6th Australian symposium**
August 23-28, Canberra, Australia
*Honorary Secretary, Miss B.J. Stevenson, PO Box 1397, Canberra City, ACT 2601, Australia*

**Laboratory '81 London**
September 8-10, London
*Curtis Steadman, 34-36 High Street, Saffron Waldon, Essex*

**Automated Analysis**
September 10-11, Stirling, UK
*Dr. C. J. Jackson, HSE, 403 Edgware Road, London NW2 6LN*

**Micro and Mini computers in the laboratory**
September 15-16, London
*Beverly Humphrey, 33-35 Bowling Green Lane, London, EC1R 0DA, UK*

**Computerised measurement**
September 22-25, Yugoslavia
*Jurema Secretariat, YU-41001 Zagreb, POB 398, Yugoslavia*

**Application of microcomputers in measurement**
September 28-October 3, Yugoslavia
*Jurema Secretariat, YU-41001 Zagreb, POB 398, Yugoslavia*

**ILMAC '81: Laboratory, chemical engineering, measurement, and automation techniques in chemistry exhibition**
September 29-October 2, Basle
*Secretariat Ilmac '81, Postfach, CH-4021 Basle, Switzerland*

**Chemasia '81**
October 21-24, Singapore
*Andrew Dedman, Chemasia '81, Clapp & Poliak Europe Ltd, 232 Acton Lane, London W4 5DL*

**1982**

**12th Annual Symposium on the Analytical Chemistry of Pollutants**
April 14-16, Amsterdam.
*Prof Dr. R.W. Frei, Congress Office, 12th Annual Symposium on the Analytical Chemistry of Pollutants, Congress Bureau, Vrije Universiteit, PO Box 7161, 1007 MC Amsterdam, The Netherlands.*

**International Congress on Automation in the Clinical Laboratory**
April 19-22, Barcelona, Spain.
*Dr. R. Galimany, Departmento de Analisis Clinicos, Seccion de Automatizacion, Cuidad Sanitaria 'Principes de Espana', Hospitalet de Llobrogat, Barcelona, Spain.*

